# Toxicity of Carbon Nanomaterials—Towards Reliable Viability Assessment via New Approach in Flow Cytometry

**DOI:** 10.3390/ijms22147750

**Published:** 2021-07-20

**Authors:** Tomáš Malina, Kateřina Poláková, Cordula Hirsch, Ladislav Svoboda, Radek Zbořil

**Affiliations:** 1 Regional Centre of Advanced Technologies and Materials, Czech Advanced Technology and Research Institute (CATRIN), Palacký University Olomouc, Šlechtitelů 27, 779 00 Olomouc, Czech Republic; radek.zboril@upol.cz; 2 Department of Physical Chemistry, Faculty of Science, Palacký University Olomouc, 17. Listopadu 12/1192, 771 00 Olomouc, Czech Republic; 3 Laboratory for Particles-Biology Interactions, Empa, Swiss Federal Laboratories for Materials Science and Technology, Lerchenfeldstrasse 5, 9014 St. Gallen, Switzerland; cordula.hirsch@empa.ch; 4 Nanotechnology Centre, Centre of Energy and Environmental Technologies, VŠB−Technical University of Ostrava, 17. Listopadu 15/2172, 708 00 Ostrava-Poruba, Czech Republic; ladislav.svoboda@vsb.cz; 5 IT4Innovations National Supercomputing Center, VŠB–Technical University of Ostrava, 17. listopadu 15/2172, 708 00 Ostrava, Czech Republic

**Keywords:** carbon nanomaterials, flow cytometry, cell viability, cytotoxicity, interference

## Abstract

The scope of application of carbon nanomaterials in biomedical, environmental and industrial fields is recently substantially increasing. Since in vitro toxicity testing is the first essential step for any commercial usage, it is crucial to have a reliable method to analyze the potentially harmful effects of carbon nanomaterials. Even though researchers already reported the interference of carbon nanomaterials with common toxicity assays, there is still, unfortunately, a large number of studies that neglect this fact. In this study, we investigated interference of four bio-promising carbon nanomaterials (graphene acid (GA), cyanographene (GCN), graphitic carbon nitride (g-C_3_N_4_) and carbon dots (QCDs)) in commonly used LIVE/DEAD assay. When a standard procedure was applied, materials caused various types of interference. While positively charged g-C_3_N_4_ and QCDs induced false results through the creation of free agglomerates and intrinsic fluorescence properties, negatively charged GA and GCN led to false signals due to the complex quenching effect of the fluorescent dye of a LIVE/DEAD kit. Thus, we developed a new approach using a specific gating strategy based on additional controls that successfully overcame all types of interference and lead to reliable results in LIVE/DEAD assay. We suggest that the newly developed procedure should be a mandatory tool for all in vitro flow cytometry assays of any class of carbon nanomaterials.

## 1. Introduction

Carbon nanostructures rank among the most promising materials in the field of nanotechnology. They include the well-known fullerenes and carbon nanotubes; however, the attention of researchers has recently shifted more towards 2D carbon nanomaterials (CNMs) and carbon dots [1,2]. As for 2D materials, the discovery of graphene in 2004 [3] was the key point, which led to the syntheses of a large number of new emerging derivatives [4]. These derivatives possess several extraordinary properties that are promising for a broad spectrum of applications [4,5]. This applies mostly to those derivatives that overcome graphene’s hydrophobicity through selective functionalization. Highly hydrophilic derivatives are especially attractive for various fields of biomedical research [4,5,6,7]. Furthermore, graphitic carbon nitrides (g-C_3_N_4_) are a new class of graphene-like materials that offer desirable optical properties similar to another type of emerging carbon nanomaterial of the last decade—carbon dots (CDs) [8,9]. Both of these materials are highly promising for applications in biosensing and bioimaging because, compared to the fluorescent inorganic semiconductors, they consist mainly of environmentally friendly elements such as carbon (C), hydrogen (H) and nitrogen (N), giving them a tremendous advantage in their biocompatibility [8,10,11,12].

The potential adverse effects of nanomaterials (NMs) on human health have been of general concern in recent years. An understanding of the cellular consequences of NMs after direct contact in vitro is the first important step and a crucial premise for their safe and successful use in biomedical applications [13]. Furthermore, the paradigm for the toxicology of the 21st century is to reliably test on the basis of high throughput in vitro cell culture-based models to minimize animal use [14,15]. One of the most important endpoints of in vitro NMs testing is acute cytotoxicity. Based on this result, it is possible to define the dose-response characteristics of nanomaterials and further focus on the understanding of the NMs’ cellular interactions with additional assays [16]. Therefore, it is crucial to have a working methodology for this endpoint to produce reliable and justified results. 

It is widely known that NMs cause interference with standard viability assays [17,18,19,20], which further highlights the need for a reliable and valid procedure for NMs in vitro testing. What is alarming is that studies referring to nanomaterial toxicity do not generally take this information into account (over 85% of papers) [18,20,21]. This fact can cause a huge problem for future safe and sustainable applications of specific nanomaterials and therefore needs to be addressed as soon as possible because different NMs can cause various types of interference [20]. Carbon nanomaterials are no exception, and interference of CNMs with common toxicity assays such as Alamar blue, neutral red, MTT and WST-1 assay has already been reported [22,23,24,25,26,27]. Therefore, for CNMs, using assays based on spectroscopic detection is recommended only with great caution. Flow cytometry is a fast and highly accurate technique providing information about individual cells in the whole population [28,29]. Thus, it represents a suitable alternative to in vitro viability testing. The LIVE/DEAD assay uses two fluorescence probes (Propidium Iodide (PI) and Calcein-AM) to distinguish between the population of dead and alive cells. PI is able to intercalate into the DNA of dead cells with a ruptured membrane, while active esterases in alive cells transform the non-fluorescent calcein-AM to highly fluorescent calcein. On the other hand, there is already a report showing interference of nanomaterials even with flow cytometry [30]. Therefore, a question should be raised whether CNMs can also cause interference with flow cytometry and, more importantly, if there is a way to overcome it.

In this study, we investigated potential interference reactions of new promising CNMs such as 2D graphene acid (GA), cyanographene (GCN), graphitic carbon nitride (g-C_3_N_4_) and 0D carbon dots (QCDs) in a basic flow cytometry assay (LIVE/DEAD). The first interference reaction was triggered by the interaction of the material with the used fluorescence probes. The second and more complicated was the interference of nanomaterials inside of the cells that caused changes in the fluorescence properties of the probes. In the case study with human skin fibroblast cells (BJ), we overcame both of these interferences and obtained reliable results for the LIVE/DEAD assay of these CNMs with a newly developed protocol using additional controls.

## 2. Results and Discussion

### 2.1. Properties of Materials

**Figure 1:**
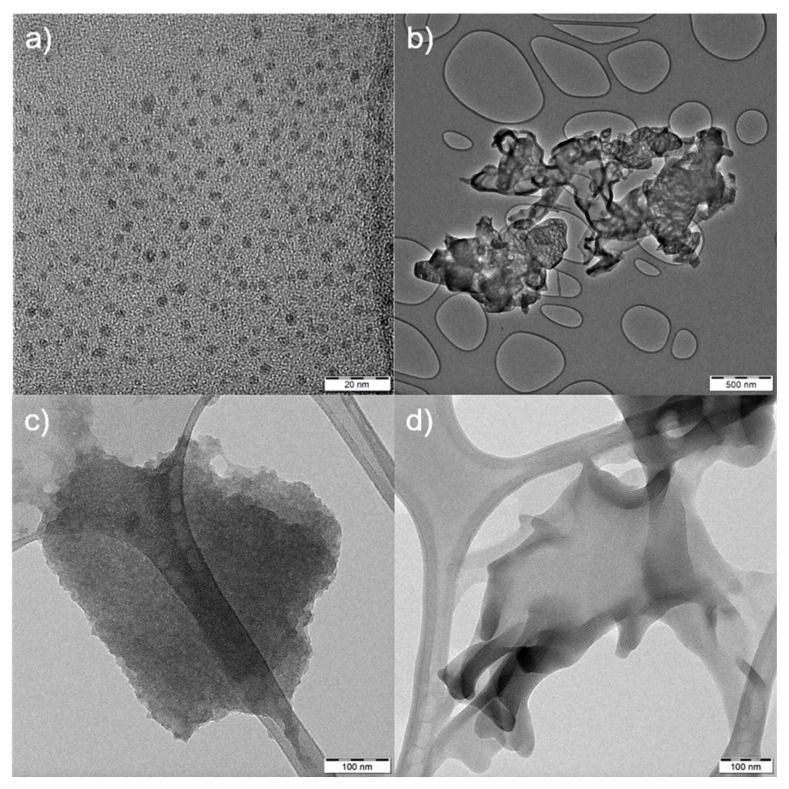
Transmission electron microscopy images of (**a**) QCDs (scale 20 nm); (**b**) g-C_3_N_4_ (scale 500 nm); (**c**) GA and (**d**) GCN (both scales 100 nm).

The four representatives of CNMs were selected to demonstrate how different properties of materials could influence their interference with in vitro testing. The size, surface charge and shape of materials are known to generally influence not only in vitro assays. The characterization of materials is summarized in Table 1. It should be noted that Table 1 only presents information given in our previous papers. GA and GCN had similar properties in the surface charge (zeta potential of −32 and −30 mV for GA and GCN, respectively), size (<500 nm according to DLS) and shape (both GA and GCN were mono/few layer sheets) (Table 1). Furthermore, neither of them exhibited fluorescence properties. On the other hand, as mentioned before, both QCDs and g-C_3_N_4_ are known for their fluorescence qualities and especially QCDs as representatives of carbon dots have wide emission spectra with strong fluorescence signals [9]. Both QCDs and g-C_3_N_4_ exhibited positive surface charge (+ 40 mV for QCDs and +24 mV g-C_3_N_4_), but they had a completely different size and shape. While QCDs were very small sphere nanodots (5 nm) [12], the g-C_3_N_4_ were loose agglomerates with irregular shapes with the Z-average of 880 nm (according to DLS) [31,32]. To have a greater awareness of the shape of NMs, we included our own TEM images, which are displayed in Figure 1.

### 2.2. Optical Microscopy Imaging and MTT Interference

The first important factor influencing possible interference of nanomaterials is their behavior in cell culture media. Although we had information about physico-chemical properties of studied nanomaterials (Table 1), these can quickly change due to the interaction with proteins present in the culture medium (precisely in FBS) [33]. Simple optical microscopy images presented in Figures S1 and S2 showed that the colloidal behavior of NMs in culture media differed greatly, depending mainly on the surface charge of carbon nanomaterials. For the positively charged g-C_3_N_4_ and QCDs, free agglomerates of NMs were observed for samples treated with 300 µg/mL (Figure S1b,c). For the g-C_3_N_4_ sample, agglomeration also occurred at a concentration of 50 µg/mL (Figure S1c). It should be noted that prior to optical microscopy imaging, the samples were washed and observed in PBS, meaning that a huge amount of free agglomerates was already washed away. When we observed the BJ cells treated with QCDs and g-C_3_N_4_ in more detail, we found that both materials at both concentrations were either internalized or attached to the membrane of the cells (Figure S2b,c). On the other hand, in the samples treated with negatively charged GA and GCN, there were fewer agglomerates seen outside of the cells (mostly only in samples treated with 300 µg/mL), but more importantly, the size of the agglomerates was much smaller than in the case of g-C_3_N_4_ and QCDs (Figure S1d,e). However, again, both materials were seen uptaken or attached to the membrane of the cells even for a concentration of 50 µg/mL and cells were completely covered with GA and GCN for the concentration of 300 µg/mL (Figure S2d,e).

At the beginning of our study, we wanted to show the inappropriateness of using the common toxicity assay for CNMs. In Figure S3, it is clearly shown that especially in the samples treated with GA and GCN, the nanomaterials could massively interfere with any type of spectrophotometric or spectrofluorometric evaluation as the supernatants are completely dark when compared to control samples. Even for the g-C_3_N_4_ and QCDs samples, the color in supernatants is different from the control samples. To assess the potential interference, we performed an MTT assay as an example. We included Blank controls (empty wells with the same treatment as for wells with cells) to try to avoid interference, as it is a standard protocol for this type of assay. From the result presented in Figure S4, it is clear that GA, GCN and g-C_3_N_4_ samples caused interference, as their MTT viability values were all below 90% of control (Figure S5). The most pronounced drop was observed for the g-C_3_N_4_ sample, as the MTT viabilities were under 50% for both concentrations, which normally indicates a huge cytotoxic effect. However, it was definitely due to the strong interference. The white color of the stock solution interfered with the blue/purple color of the dissolved formazan crystals because the microscopy imaging showed almost no dead or damaged cells (Figures S1 and S2). Additionally, Figure S4 shows that cells treated with the g-C_3_N_4_ sample were much brighter than the untreated control. For the GA and GCN samples, we again observed almost no dead cells in microscopy images (Figures S1 and S2). On the other hand, we saw many NMs either covering the cells’ surface or being inside of the cells, which could potentially cause a decrease in the MTT signal through interaction with formazan crystals, as was already reported for other CNMs [25,27]. We could not determine any interference for the QCDs sample, as all the values were similar to the untreated control and there was no change observed in the color (Figures S4 and S5).

### 2.3. Interference of CNMs in Forward and Side Scatter Profiles in Flow Cytometry

**Figure 2:**
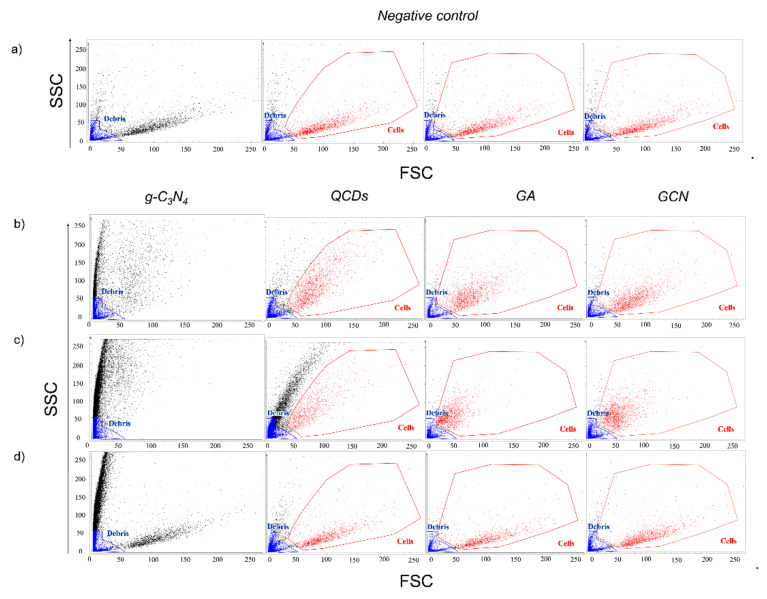
Forward and side scatter profiles for samples: (**a**) negative control of BJ cells (4× same sample with specific gates for each NMs); BJ cells treated for 24 h with (**b**) 50 µg/mL and (**c**) 300 µg/mL of carbon nanomaterials and (**d**) spike-in controls (from left to right: g-C_3_N_4_, QCDs, GA and GCN). The population of cells is highlighted in red, the population of debris is marked blue and the remaining black population in samples g-C_3_N_4_ and QCDs is considered NMs agglomerates.

Different colloidal behavior of CNMs in culture media was confirmed by flow cytometry in the forward and the side scatter profiles as well (Figure 2). The extra population of events, besides populations of cells and debris, occurred only in those samples treated with g-C_3_N_4_ and QCDs, which can be solely assigned to agglomerated NMs (Figure 2b,c). We proved this hypothesis by using spike-in controls, the first crucial controls that have to be definitely used, where a similar population of events was observed especially for the g-C_3_N_4_ and slightly also for the QCDs sample (Figure 2d). Spike-in controls are meant to mimic the highest possible concentration of free NMs in samples by adding the appropriate volume of NMs stock solutions in water to the negative control sample right before the measurement. There was also a clear shift in the cells’ population scatter profile compared to the untreated sample, which indicates either internalization or membrane attachment of both g-C_3_N_4_ and QCDs samples. Even though we did not see for the samples treated with GA and GCN any events representing free agglomerates in scatter profiles (including spike-in controls), the cells’ population shifted again greatly, compared to the untreated sample (Figure 2). These results agreed well with observations from optical microscopy images.

As it is known from the literature [34], positively charged NMs show significantly higher interaction with proteins (especially albumin and globulins that are present in FBS) than negatively or neutral charged NMs. The reason behind this phenomenon is most probably the fact that the most abundant proteins in FBS (albumin and globulins) carry a negative net charge at physiological pH [35]. Therefore, a nanomaterial with positive zeta potential would preferentially interact with such proteins. It was already reported that the zeta potential of NMs with the protein corona is negative in most cases, which further supports the hypothesis of higher adsorption of proteins on NMs with a positively charged surface [35,36]. Regarding g-C_3_N_4_, it was understandable that the interaction of large positively charged NMs with proteins resulted in the formulation of agglomerates of a size significant enough to be seen in microscopy images and scatter profiles (Figure 2, Figures S1 and S2). Surprisingly, even 5 nm QCDs produced agglomerates with proteins big enough to be detected by those techniques. As mentioned before, one of the reasons is definitely the strong positive surface charge of QCDs (+40 mV) resulting in a massive interaction with proteins. However, as Glancy et al. recently reported, the protein corona of sub-10 nm nanoparticles is more complex and nanoparticles can serve more as cargo on a protein rather than as a carrier of the protein, as is usually the case of larger NMs [37]. Hence, the agglomerates of QCDs in the culture medium could be a mix of several nanoparticles in combination with various proteins, which could explain the size of those agglomerates.

### 2.4. Interference of CNMs in Spike-in Controls

Having in mind a formation of agglomerates of g-C_3_N_4_ and QCDs in culture media these CNMs are famous for their extraordinary fluorescence properties [12,32]. Logically, it was crucial to check if those agglomerates could interfere with the fluorescence probes (PI, Calcein) used in the LIVE/DEAD assay. This was carried out using spike-in controls again.

**Figure 3:**
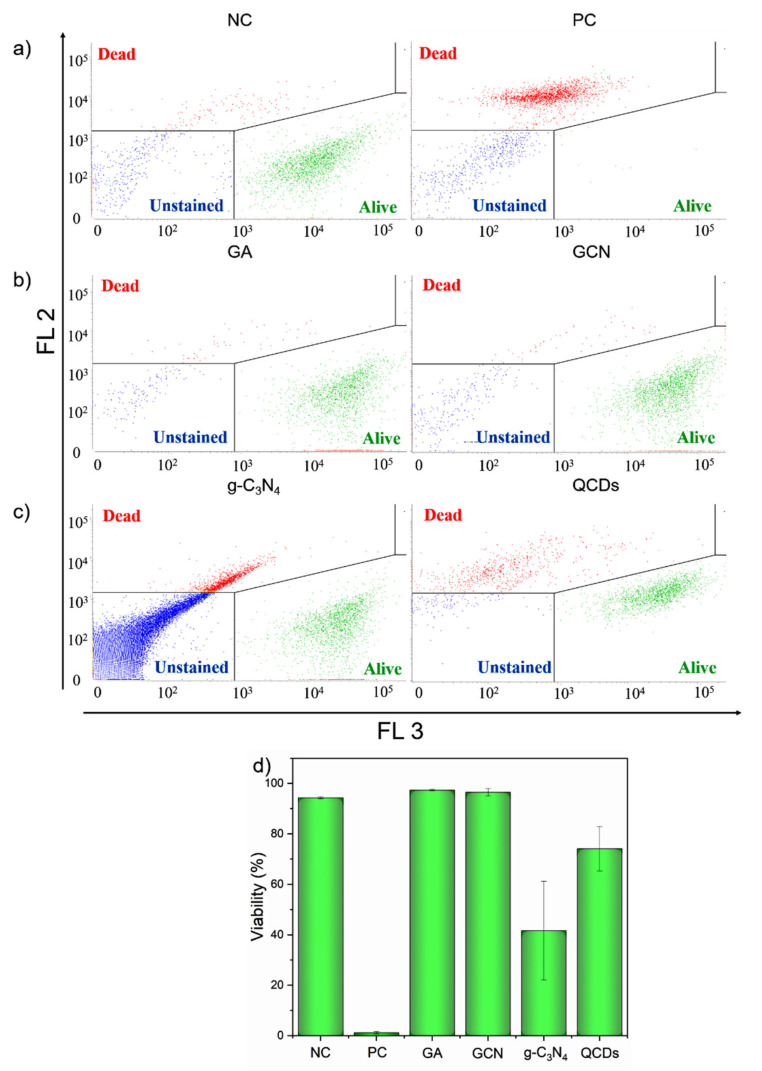
Dot plot showing LIVE/DEAD assay of: (**a**) negative and positive control of BJ cells; (**b**) BJ cells spike-in controls for GA (**left**) and GCN (**right**) and (**c**) BJ cells spike-in controls for g-C_3_N_4_ (**left**) and QCDs (**right**). Gates were selected according to the NC and PC samples. (**d**) The evaluation of the viability of spike-in controls’ samples (*n* = 3). The events in the *alive* gate are shown in green, events in the *dead* gate in red and events in the *unstained* gate are highlighted in blue.

First, we measured the spike-in controls for all NMs and analyzed them in a dot plot of red channel (FL 2: ex. 488/em. 700 nm) for PI-positive cells against green channel (FL 3: ex. 488/em. 527 nm) of calcein positive cells. To see if the free NMs can interfere with the fluorescence probes, we gated out the population of debris shown in Figure 2 (blue population of events). The dot plots of the LIVE/DEAD assay are shown in Figure 3. The first important thing to observe is that even after we discarded the debris population of events, there were still some events of unstained cells (*unstained* gate) with the fluorescence intensity in both channels lower than 10^3^ in the log scale in negative and positive control samples (Figure 3a). This was not included in the evaluation of viability. Then, we created gates according to the positive and negative control samples for dead (red, PI-positive) and alive (green, calcein positive) cells (Figure 3a) and used them for evaluation of the viability of spike-in controls for NMs (Figure 3d). As expected for the negatively charged GA and GCN, no population of free agglomerates of NMs was present to interfere with the results (Figure 2d, Figure 3b and Figure S7b), as the viability of spike-in controls for both of these materials remained over 90%, similar to the negative control (Figure 3d). However, for positively charged g-C_3_N_4_ and QCDs, an additional population of events appeared in the scatter profiles (Figure 2d) as well as in the dot plots (Figure 3c and Figure S7c). This population represents the agglomerates of NMs because the only difference between those samples and the negative control (or positive control in the case of spike-in PC samples) was the addition of NMs right before the measurement. The spike-in control for g-C_3_N_4_ generated a huge number of agglomerates, comparable to the amount we saw in the sample after 24 h of treatment (Figure 2c,d). Furthermore, the agglomerates interfered greatly with the evaluation as the viability of the g-C_3_N_4_ spike-in sample dropped to 42% (Figure 3d). On the other hand, in spike-in control for QCDs, the amount of agglomerates was significantly lower than after 24 h (Figure 2c,d). This is most probably due to the difference in the size of g-C_3_N_4_ and QCDs because the formulation of agglomerates takes more time with ultra-small nanoparticles and the spike-in controls are measured immediately after adding the NMs. However, even a lower amount of agglomerates was responsible for the decrease in viability of the QCDs spike-in control sample to 74% (Figure 3d).

Spike-in controls are a necessary tool in flow cytometry and should be used in every measurement, where any interference of NMs is expected. Only when spike-in controls are used, gating—the most important step in flow cytometry—is performed properly. It is crucial that we have information only about the population of cells in the sample. Nevertheless, spike-in controls cannot give information about cells’ scatter profiles, characterizing the cell size and granularity (complexity) [38]. Of course, the size and granularity of cells are influenced after 24 h of treatment with NMs. However, this is not the case with spike-in controls as the NMs are added just before the measurement.

Even though spike-in controls helped massively with choosing the specific gates correctly (Figure 2), they did not give us any information about the behavior of NMs inside the cells or on their membrane. Given that all NMs somehow influenced the cells’ scatter profiles (Figure 2), we needed to introduce another important type of control named nanomaterial positive control (NM PCs). In these controls, we mimicked the situation where all the cells treated with NMs for 24 h would be dead to get information about the profile of dead cells with NMs on their membrane or inside. We heat-killed the cells treated with NMs for 24 h before the measurement. We did not need any control for alive cells treated with NMs for 24 h as, according to the optical microscopy, the majority of the cells were alive in all samples (Figure S2).

In the next chapters, we will give detailed information on how it is possible to select correct gating, avoiding different types of interference, and get reliable results in LIVE/DEAD assay for all four types of our chosen CNMs following our newly developed approach using a combination of both additional controls. For a standard procedure, we applied the well-known method of gating using negative (over 90% viability) and positive control (under 10% viability). In our new approach, gates were set as follows: viability of negative and spike-in controls over 90%, the viability of positive and nanomaterial positive controls (NM PCs) under 10%.

### 2.5. Interference of QCDs in LIVE/DEAD Assay

As it is seen in Figure 2, for samples treated with QCDs, there was a clear change in the scatter profiles and another population of agglomerates emerged for the samples treated with 50 µg/mL and especially 300 µg/mL. That is why it was quite challenging to gate only the population of cells as agglomerates might also intermingle to some extent. Using the spike-in control, we could observe the profile of free agglomerates in the dot plot of red against green channels (Figure 3c and Figure S7c). Therefore, when there were almost no events in the dot plot of the sample after 24 h, which correlated with the free agglomerates in the dot plot of spike-in control, we knew we had the correct gate, as it displayed the population of cells with as few agglomerates as possible (Figure 2). With this gating, we performed LIVE/DEAD assay evaluation using dot plots of two fluorescent channels.

**Figure 4:**
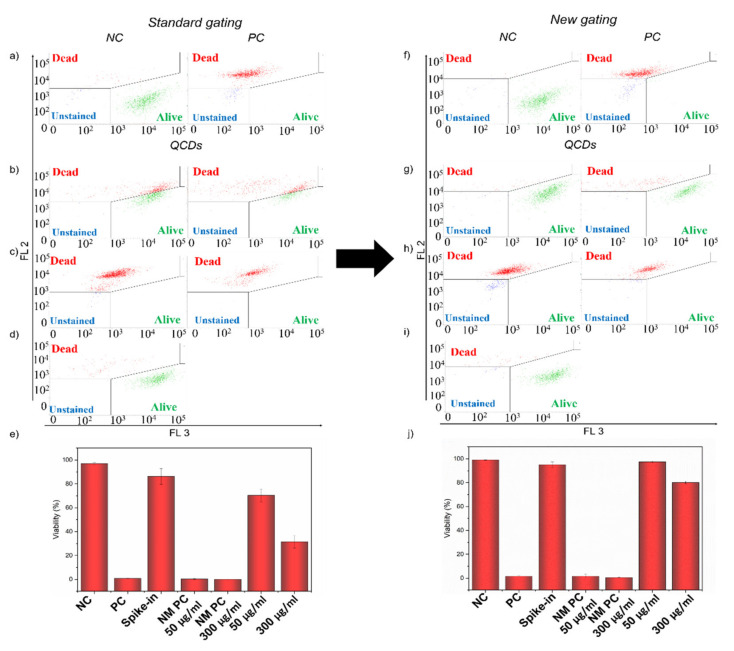
Dot plot showing LIVE/DEAD assay of: (**a**,**f**) negative and positive control of BJ cells and (**b**–**d**,**g**–**i**) BJ cells treated with QCDs samples with (**a**–**d**) standard gating and (**f**–**i**) new gating approach. (**b**,**g**) Samples treated with 50 µg/mL (**left**) and 300 µg/mL (**right**) of QCDs for 24 h; (**c**,**h**) Samples treated with NM PC 50 µg/mL (**left**) and NM PC 300 µg/mL (**right**); (**d**,**i**) Spike-in control for QCDs. (**e**,**j**). The evaluation of the viability of BJ cells treated with QCDs and additional control samples (*n* = 3). The events in the *alive* gate are shown in green, events in the *dead* gate in red and events in the *unstained* gate are highlighted in blue.

First, we analyzed the samples with standard gating using only negative and positive control. From Figure 4a–e, it is clear that there was the interference of optical properties of QCDs [12,31,32] even though the viability of NM PCs was under 10%, which was correct for heat-killed cells (Figure 4e). Interaction of QCDs with cells resulted in the shift in the fluorescence intensity in the red detector (FL 2: ex. 488/em. 700 nm), which was strong enough to move some of the cells from the *alive* gate to the *dead* gate (Figure 4b). Additionally, although we carefully tried to gate only the population of cells (Figure 2), there were still some events representing free agglomerates left in the samples, as the viability of spike-in control for QCDs decreased to 86% (Figure 4d,e). Therefore, there was an interference with the evaluation as the fluorescence shift and the presence of agglomerates in gating according to the control samples resulted in decreasing the viability to 70 and 32% for samples treated with 50 and 300 µg/mL of QCDs, respectively (Figure 4e).

Thus, there were two challenges for the LIVE/DEAD assay of BJ cells treated with QCDs. First was the influence of the optical properties of QCDs, and, second was the presence of free agglomerates in culture media that were not gated out of evaluation. To show how to overcome these challenges, we analyzed the same dataset with a new gating protocol according to the additional controls, which is displayed in Figure 4f–j. Using these gates, the whole population of alive cells remained in the *alive* gate despite the fluorescence shift (Figure 4g) and the events representing free agglomerates were now in the *unstained* gate, and were not included in the evaluation (Figure 4i). This setup resulted in 98 and 80% of the viability of BJ cells after 24 h of incubation with 50 and 300 µg/mL of QCD, respectively (Figure 4j).

Therefore, due to the interference in the analysis according to standard gating, QCDs could be falsely considered toxic to BJ cells, although the reliable viability of BJ cells did not drop under 80% even for concentration 300 µg/mL of QCDs.

### 2.6. Interference of g-C_3_N_4_ in LIVE/DEAD Assay

For the g-C_3_N_4_ sample, even before the LIVE/DEAD assay itself, we observed another challenge. The interaction of cells with 50 µg/mL and especially 300 µg/mL of g-C_3_N_4_ caused the side scatter values of these samples to shift out of the defined scale (Figure 2). We have not observed this phenomenon with any other material before. As for the right evaluation, we needed only the population of cells (parameters of measurement were set according to the controls and those cannot be changed during the measurement). That is why we used a dot plot profile of forward scatter values against values of the fluorescent channel (FL–1: ex. 405/em. 528 nm) for gating the population of cells where we did not expect any increase in the fluorescence intensity for calcein and PI (Figure S6). Even with the use of another detector, there was an overlap between some events of the populations of free NMs and events from the population of cells (Figure S6b). We used the same procedure as we did for the QCDs samples to try to gate only the population of cells without any free agglomerates of g-C_3_N_4_ (Figure S6a). However, at a concentration of 300 µg/mL, some of g-C_3_N_4_ free agglomerates had to be gated as well as there was no clear line that would separate them from the cells (Figure S6b).

**Figure 5:**
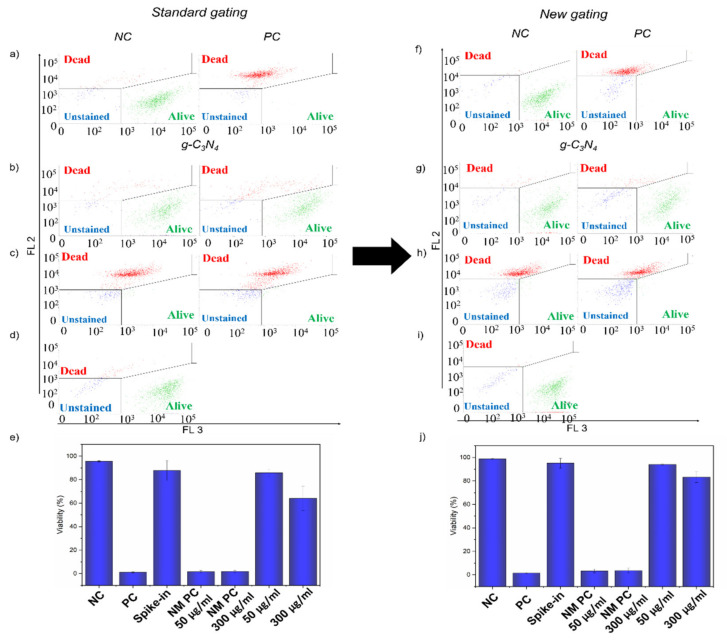
Dot plot showing LIVE/DEAD assay of: (**a**,**f**) negative and positive control of BJ cells and (**b**–**d**,**g**–**i**) BJ cells treated with g-C_3_N_4_ samples with (**a**–**d**) standard gating and (**f**–**i**) new gating approach. (**b**,**g**) Samples treated with 50 µg/mL (**left**) and 300 µg/mL (**right**) of g-C_3_N_4_ for 24 h; (**c**,**h**) Samples treated with NM PC 50 µg/mL (**left**) and NM PC 300 µg/mL (**right**); (**d**,**i**) Spike-in control for g-C_3_N_4_. (**e**,**j**) The evaluation of the viability of BJ cells treated with g-C_3_N_4_ and additional control samples (*n* = 3). The events in the *alive* gate are shown in green, events in the *dead* gate in red and events in the *unstained* gate are highlighted in blue.

In the standard gating, even though the fluorescence intensity of g-C_3_N_4_ was not as distinctive as for QCDs, there was a shift in some cells from the *alive* to the *dead* gate in the sample treated with 300 µg/mL (Figure 5b). A more serious problem was free agglomerates that were gated in the population of cells, as was mentioned above, and were now presented in the *dead* gate. This was pronounced in the spike-in control g-C_3_N_4_ sample, which had 88% viability (Figure 5d,e). Although the decrease in viability was slighter than for QCDs, it was another indication that there was interference in the sample. Furthermore, the events presented in Figure 5d showed a dot plot profile comparable to the one in the spike-in control for g-C_3_N_4_ displayed in Figure 3c. Nevertheless, because of the mentioned overlap of agglomerates with cells in the samples after 24 h (Figure S6), we had to include them, as we could not afford to lose information about those cells. Thus, due to the standard gating according to the positive and negative control, events representing free agglomerates in the *dead* gate were responsible for the drop in the viability of samples treated with 50 and 300 µg/mL of g-C_3_N_4_ to 86 and 63%, respectively (Figure 5e).

By applying new gating according to additional controls presented in Figure 5f–j, we managed to avoid this interference. Using specific gates in the LIVE/DEAD dot plot, we evaluated only the population of cells. Events representing free agglomerates of g-C_3_N_4_ were now in the *unstained* gate and the spike-in control viability was over 90% (Figure 5i,j). Therefore, the reliable results of the viability of BJ cells after 24 h of incubation were 94 and 83% for 50 and 300 µg/mL of g-C_3_N_4_, respectively (Figure 5j).

### 2.7. Interference of GA and GCN Samples in LIVE/DEAD Assay

**Figure 6:**
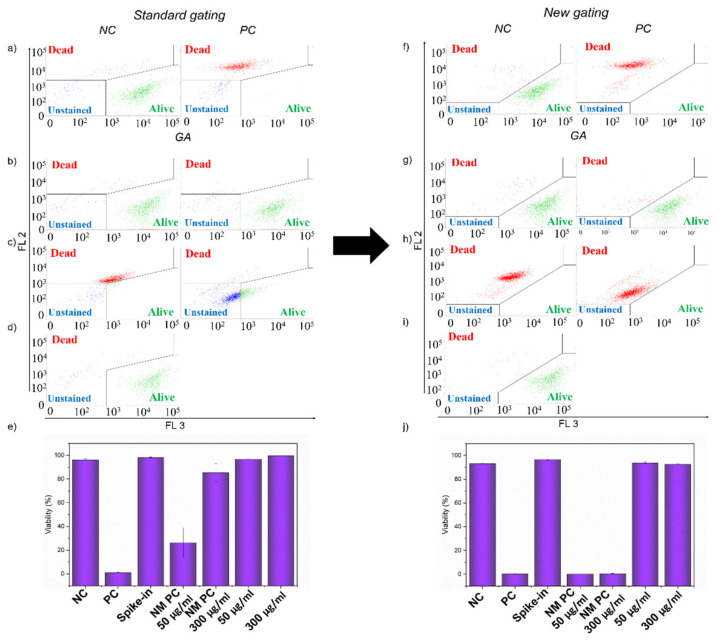
Dot plot showing LIVE/DEAD assay of: (**a**,**f**) negative and positive control of BJ cells and (**b**–**d**,**g**–**i**) BJ cells treated with GA samples with (**a**–**d**) standard gating and (**f**–**i**) new gating approach. (**b**,**g**) Samples treated with 50 µg/mL (**left**) and 300 µg/mL (**right**) of GA for 24 h; (**c**,**h**) Samples treated with NM PC 50 µg/mL (**left**) and NM PC 300 µg/mL (**right**); (**d**,**i**) Spike-in control for GA. (**e**,**j**). The evaluation of the viability of BJ cells treated with GA and additional control samples (*n* = 3). The events in the *alive* gate are shown in green, events in the *dead* gate in red and events in the *unstained* gate are highlighted in blue.

Both GA and GCN samples formed much smaller agglomerates in culture media, which did not interfere with gating in the scatter profiles, as the population of cells was still clearly distinguishable (Figure 2). On the other hand, there was still a notable shift in scatter profiles, and optical microscopy showed the cells either filled with NMs or with NMs attached to the membrane after 24 h of incubation (Figure 2 and Figure S2). Therefore, there was a high probability that both materials would interfere with the dyes of the LIVE/DEAD assay. As the interference was the same for both materials, we used a similar methodology including gating to avoid it. That is why only the example of GA is presented in Figure 6, while the results of GCN were moved to SI (Figure S8).

The evaluation with standard gating displayed in Figure 6a–e confirmed the interference of the GA sample. However, it was a completely different type of interference than the one observed for QCDs and g-C_3_N_4_ materials. There was a decrease in the fluorescence intensity of PI in the red channel (FL 2) caused by a quenching effect of the GA sample. This phenomenon was clearly manifested in NM PCs. While events representing dead cells in positive control showed the fluorescent intensity of around 10^4^ in the FL 2 channel (Figure 6a), there was a slight decrease even in the events’ intensity for the NM PC 50 µg/mL (fl. intensity between 10^3^ and 10^4^, Figure 6c). For the NM PC 300 µg/mL sample, the reduction was distinctly more pronounced as events representing the dead cells showed fluorescent intensity values of around 10^2^ (Figure 6c). Therefore, although samples treated with 50 µg/mL and 300 µg/mL had viability over 96%, the evaluation was not reliable as the quenching effect of the GA sample towards PI caused the events representing the dead cells to drop from the *dead* gate to the *alive* or *unstained* gate (Figure 6b,c,e). This interference led to a false increase in the viability of NM PCs to 26% and 85% for the NM PC 50 µg/mL and the NM PC 300 µg/mL, respectively, when standard gating was applied (Figure 6e). For GCN, the quenching effect was even more profound as the viability of NM PC 300 µg/mL was 95% (Figure S8e).

Through a new gating procedure according to the additional controls, we managed to avoid the negative influence of quenching, which resulted in the viability of NM PCs samples dropping below 10% (Figure 6h,j). We thus knew that even with the quenching of GA, events representing dead cells would be in the *dead* gate in the samples treated with 50 µg/mL and 300 µg/mL of GA (Figure 6g). Therefore, reliable viabilities of the BJ cells treated for 24 h with 50 µg/mL and 300 µg/mL of GA were 94 and 92%, respectively (Figure 6j). The treatment with GCN resulted in viabilities of 96 and 93% for 50 µg/mL and 300 µg/mL, respectively (Figure S8j).

## 3. Materials and Methods

### 3.1. Materials and Characterization

For this study, we have chosen four emerging CNMs: (1) new graphene derivatives graphene acid and cyanographene, (2) positive carbon dots with quaternary ammonium groups on their surface, and (3) exfoliated carbon nitride. Details about the synthesis and characterization of the mentioned materials can be found in the following papers: GA and GCN [7], QCDs [12] and g-C_3_N_4_ (material labeled as NS500 [31] and NS [32]). Samples were also characterized by transmission electron microscopy (TEM, JEOL 2100 operating at 160 kV).

### 3.2. Cell Culture

Human skin fibroblasts BJ (ATCC, CRL-2522) were used for this study. Cells were cultivated at 37 °C under a 5% CO_2_ atmosphere in EMEM—Eagle’s Minimum Essential Medium (Sigma Aldrich, St. Louis, MI, USA) supplemented with (final concentrations in medium): L-Glutamine (2 mM), Non-essential amino acids (NEAA, 1x), fetal bovine serum (FBS, 10%), PenStrep (5 U penicillin, 50 µg streptomycin/mL) and sodium bicarbonate (2 g/L).

### 3.3. Flow Cytometry Scatter Profiles and LIVE/DEAD Assay

The LIVE/DEAD assay was performed using a BD FACSVerse flow cytometer (BD Biosciences, San Jose, CA, USA). First, the BJ cells were seeded into a 96-well plate at a density of 10,000 cells/well. Then we treated the cells with 50 and 300 µg/mL of GA, GCN, QCDs and g-C_3_N_4_ diluted in 100 µL of culture media and incubated for 24 h. After 24 h, we collected the supernatant (100 µL), washed the cells with phosphate-buffered saline (PBS; 0.1 M, pH 7.4; 25 µL), detached the cells with 0.25% trypsin-EDTA solution (Sigma Aldrich; 25 µL), and, after 5 min, we resuspended the cells in 150 µL of a culture medium (final volume 300 µL). There was no volume discarded during the preparation (even PBS for washing was collected), so we obtained information about all cells in our samples. Then, the cells were incubated with PI (10 µg/mL) and calcein-AM (50 µM) diluted in a culture medium for 30 min in the dark. Finally, the fluorescence signal was measured on a flow cytometer using the first two scatters (Forward scatter channel vs. Side scatter channel intensity using linear scale—0, 50, 100, 150, 200, 250 RFU) and then two fluorescence channels (red FL 2: ex. 488/em. 700 nm and green FL 3: ex. 488/em. 527 nm intensity using logarithmic scale—0, 10^2^, 10^3^, 10^4^, 10^5^ RFU). Heat-killed cells that were incubated at 60 °C for 30 min before measurements were used as a positive control. As we worked with materials with fluorescence properties, we turned off automatic compensation and compensated fluorescence channels two and three manually. Three independent experiments were performed and mean and standard deviation ± (SD) were calculated.

### 3.4. Flow Cytometry Controls

As there was an interference of carbon nanomaterials with the flow cytometry measurement, the use of only one positive control was not enough. That is why we used two types of additional controls. The first type of control was called spike-in control and was based on a type that was already presented in Bohmer et al. [30]. Because of the particular cell harvesting procedure that collects everything (supernatants, PBS, cells) in the same tube, the nanomaterials were present in the staining solution. Therefore, we added the same volume of stock solutions, equaling the highest concentration (300 µg/mL) right before the measurement in order to mimic the situation where no NMs were uptaken and ended up free in the staining solution, potentially interfering with free fluorescence probes. The second type of control was named nanomaterial positive control (NM PC). The preparation of the NM PCs was the same as for the cells treated with NMs, but the NM PCs were heat-killed (60 °C for 30 min) right before the measurement in the same way as the positive control. This way, we obtained information about the possible interference of internalized NMs with fluorescence probes.

## 4. Conclusions

In this study, we showed that carbon nanomaterials caused different types of interference in flow cytometry LIVE/DEAD assay, and correct gating was identified as a crucial step in the evaluation of real viability. We carefully performed gating in scatter profiles to display only the population of cells, along with specific gating in a LIVE/DEAD dot plot distinguishing between the alive and dead population of cells. When a standard gating procedure according to negative and positive control was applied, CNMs’ optical properties, together with the ability to form agglomerates and the quenching of fluorescence of commercial probes, led to false results. Here, we demonstrated how to overcome CNMs’ interference using a new gating procedure with careful selection of specific gates according to spike-in and nanomaterial positive controls. Our reliable approach improved the false toxic effect caused by interference by 28 and 48% for QCDs (50 and 300 µg/mL, respectively) and by 8 and 20% for g-C_3_N_4_ (50 and 300 µg/mL, respectively). Moreover, our procedure overcame the quenching effect and correctly decreased the viability of dead cells in NM PC 50 µg/mL NM PC 300 µg/mL by 26 and 84% for GA and by 22 and 92% for GCN.

## Figures and Tables

**Table 1:** Characterization of carbon nanomaterials g-C3N4, QCDs, GA and GCN.

	g-C_3_N_4_ [31,32]	QCDs [12]	GA [7]	GCN [7]
Size (DLS, nm)	880	5	200	300
Zeta potential (mV)	+24	+40	−32	−30
Shape	loose agglomerates with irregular shape	sphere	mono/few layer sheets	

## Data Availability

All data from this paper are available upon reasonable request to the corresponding author.

## Supplementary Materials

The following are available online at https://www.mdpi.com/article/10.3390/ijms22147750/s1.
