# Supplementary material for: Toxicity of Carbon Nanomaterials—Towards Reliable Viability Assessment via New Approach in Flow Cytometry
Source: Int J Mol Sci. 2021 Jul 20;22(14):7750. doi: 10.3390/ijms22147750 (PMC8305450; doi:10.3390/ijms22147750)
Supplement: Supplementary file 1 [file ijms-22-07750-s001.zip › ijms-1223210-supplementary.pdf]

Supplementary material

for

Toxicity of carbon nanomaterials - towards reliable viability assessment via new approach in flow cytometry

Tomáš Malina[1,2,*], Kateřina Poláková[1,*], Cordula Hirsch[3], Ladislav Svoboda[4,5] and Radek Zbořil[1,4]

[1]Regional Centre of Advanced Technologies and Materials, Czech Advanced Technology and Research Institute, Palacký University, Křížkovského 511/8, 779 00 Olomouc, Czech Republic

[2]Department of Physical Chemistry, Faculty of Science, Palacký University Olomouc, Křížkovského 511/8, 779 00 Olomouc, Czech Republic

[3]Laboratory for Particles-Biology Interactions, Empa, Swiss Federal Laboratories for Materials Science and Technology, Lerchenfeldstrasse 5, 9014 St. Gallen, Switzerland

[4]Nanotechnology Centre, Centre of Energy and Environmental Technologies, VŠB−Technical University of Ostrava, 17. Listopadu 2172/15, 708 00 Ostrava-Poruba, Czech Republic

[5]IT4Innovations, VŠB – Technical University of Ostrava, 17. listopadu 15/2172, Ostrava 708 00, Czech Republic

[*]Corresponding authors: Tomáš Malina, Kateřina Poláková

Email address: tomas.malina@upol.cz, katerina.polakova@upol.cz

Number of Figures: 8

Number of Pages: 12

Supporting Text and Figures

Optical microscopy of BJ cells treated with carbon nanomaterials

For optical microscopy, we seeded BJ cells in a 24-well plate (50 x 10^3 cells/well), treated them with the same NMs' concentrations and incubated them again for 24 h. The imaging was performed using IX70 optical microscope (Olympus, Japan). Before the measurement, we washed the cells twice with 0.5 ml PBS, added fresh 0.5 ml PBS and took optical microscopy images. First, under lower magnification (10x objective), to obtain information about a higher amount of cells in the samples (Figure S2), and then, under higher magnification, (40x objective) to get details about NMS internalization (Figure S3).

a)

b)

c)

d)

e)

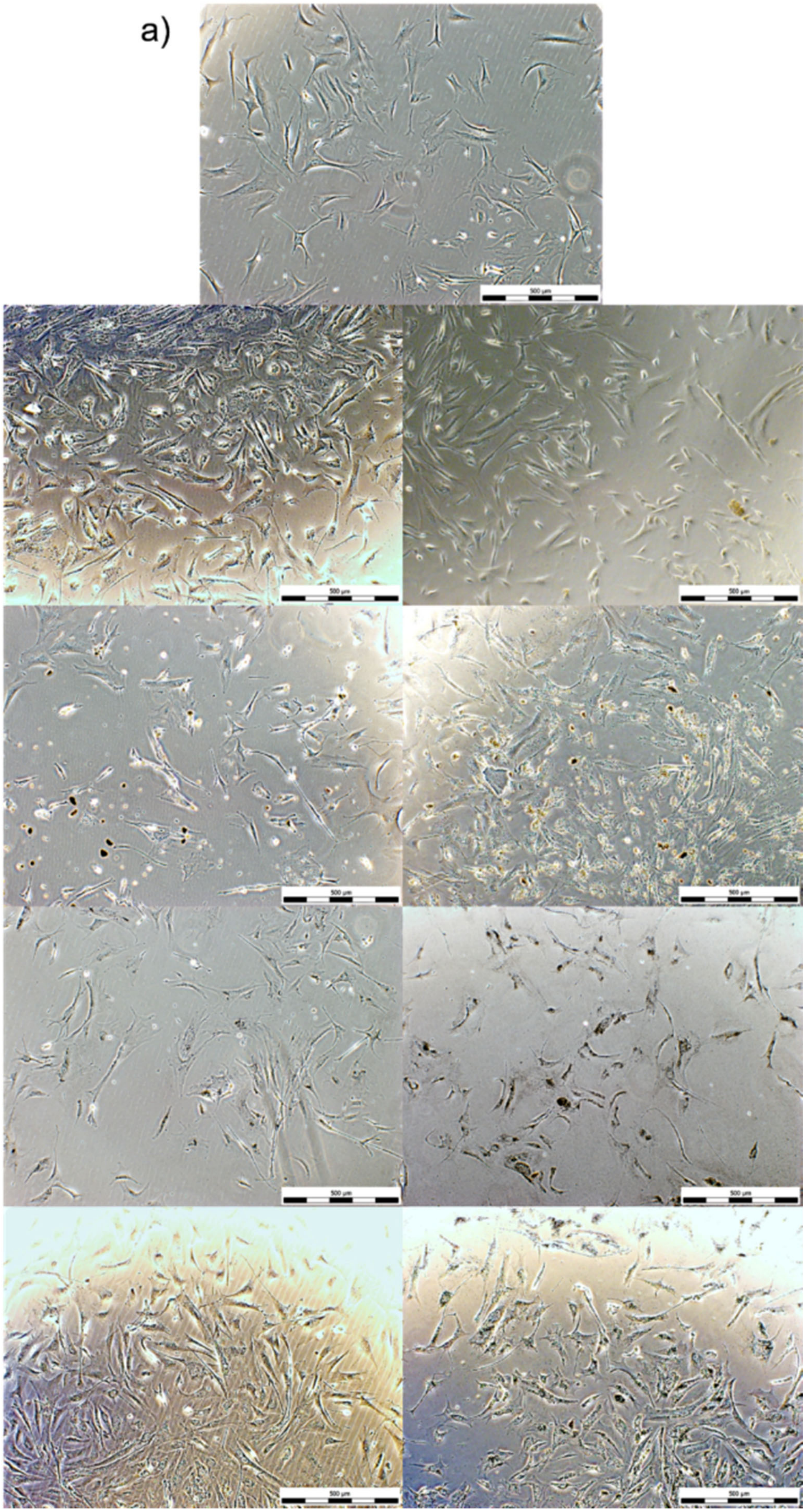

Figure S1. Optical microscopy images of a) untreated BJ cells in PBS after removal of supernatant. Images of BJ cells treated for 24 h with: b) QCDs, c) g-C_3N_4, d) GA and e) GCN (concentration 50 μg/ml (left) and 300 μg/ml (right)). The scale bar is 500 μm.

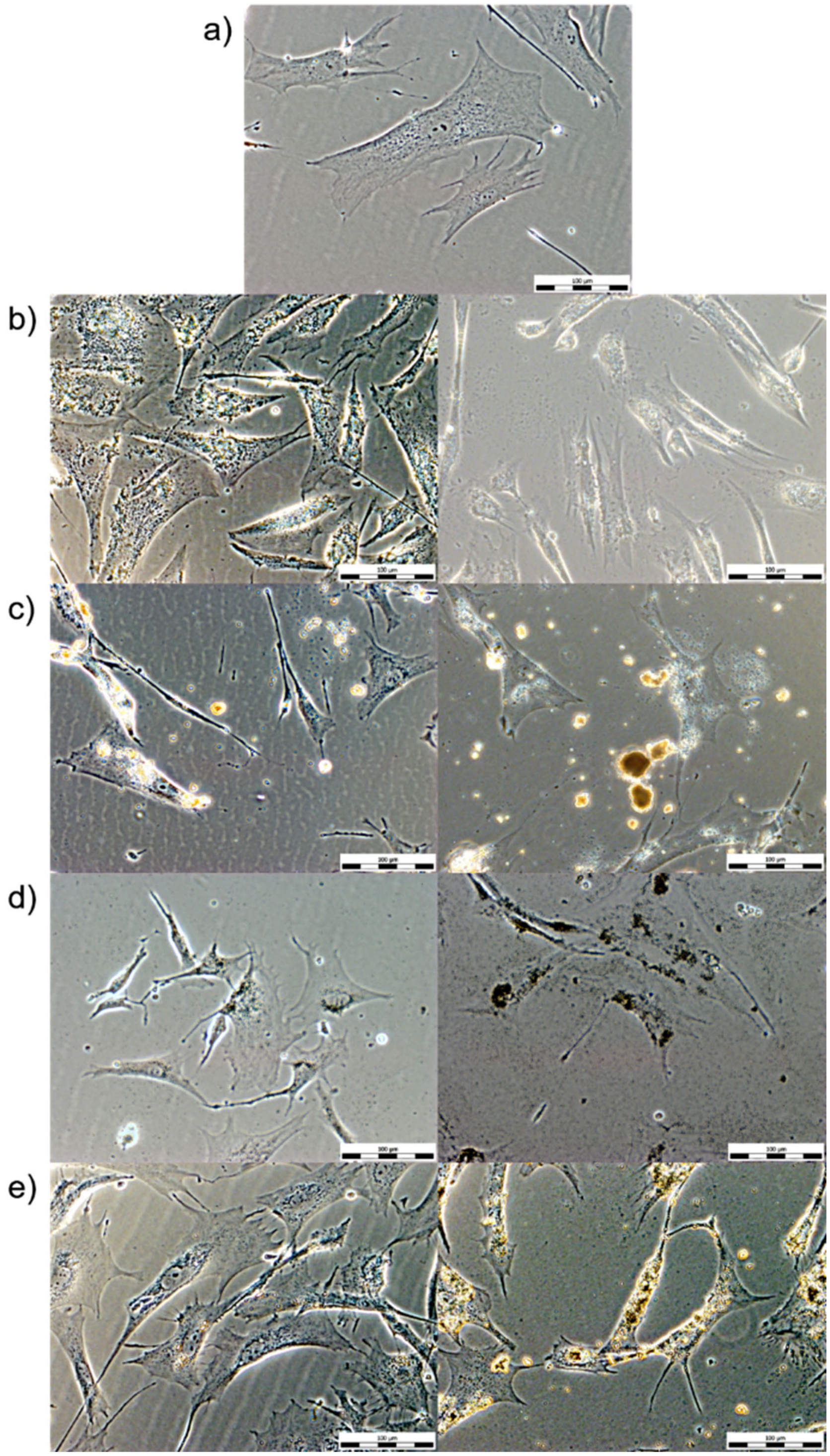

Figure S2. Optical microscopy images of a) untreated BJ cells in PBS after removal of supernatant. Images of BJ cells treated for 24 h with: b) QCDs, c) g-C_3N_4, d) GA and e) GCN (concentration 50 µg/ml (left) and 300 µg/ml (right)). The scale bar is 100 µm.

Interference of CNMs with MTT assay

To display an example of interference of CNMs with common viability assays, we chose the MTT assay as one of the most frequently used in literature. We treated 10 x 10^3 BJ cells seeded in 96-well plate with 50 and 300 µg/ml of our materials for 24 h. Plate layout after 24 h is displayed in Figure S3). Negative controls (NC) received a complete cell culture medium only. Discoloration (blackening) of the supernatants of GA, GCN as well as, to a lesser extent, QCD treated samples can readily be seen with the naked eye. After the treatment, we discarded the supernatant, washed the cells once with PBS (100 µl) and added the MTT solution diluted in culture media (final concentration 0.5 mg/ml). After 4 h of incubation, we discarded the supernatant and added 100 µl of DMSO. Then we shook the plate for 10 minutes and measured absorbance of 570 nm (Figure S4). We included blank controls in the measurement, which were empty wells treated for 24 h with 50 and 300 µg/ml of CNMs, to check how sedimented materials can interfere with the MTT assay (Figure S3). In the following analysis, we subtracted the values of blank controls from the values of the treated cells, and the result is displayed in Figure S4.

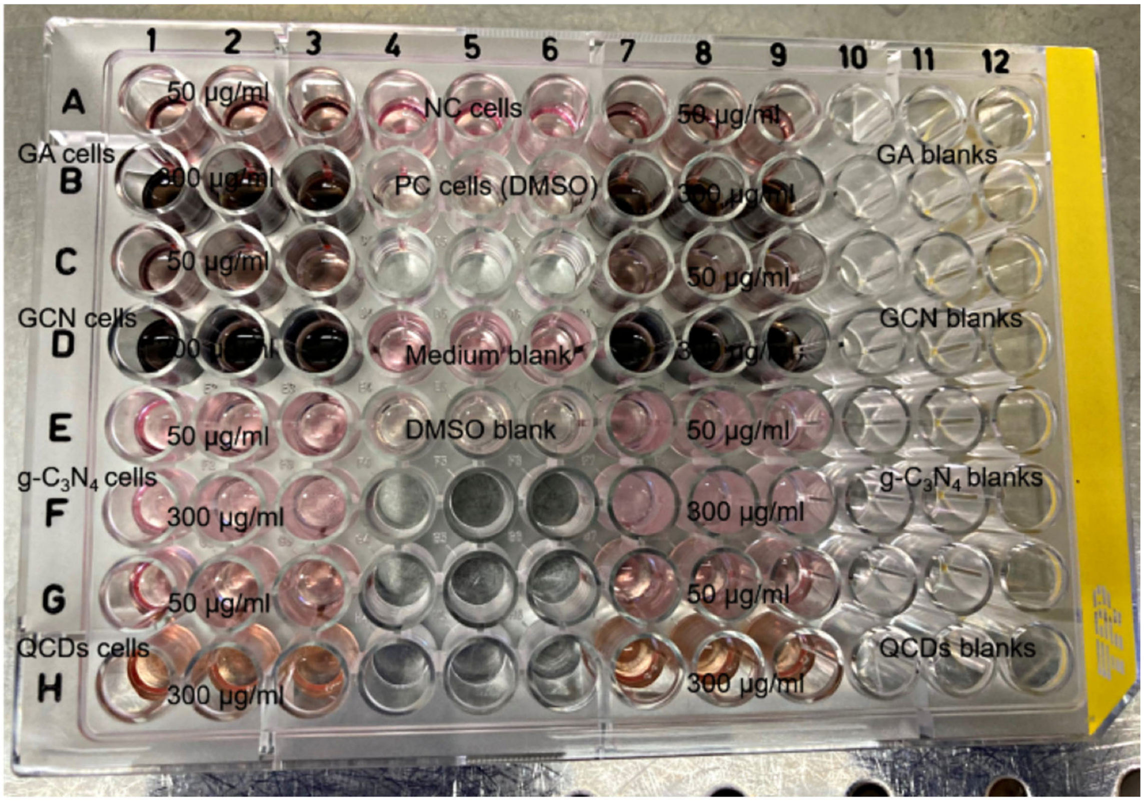

Figure S3. Image of the whole 96-well plate of BJ cells after 24 h incubations with 50 and 300 μg/ml of carbon nanomaterials (g-C_3N_4, QCDs, GA and GCN).

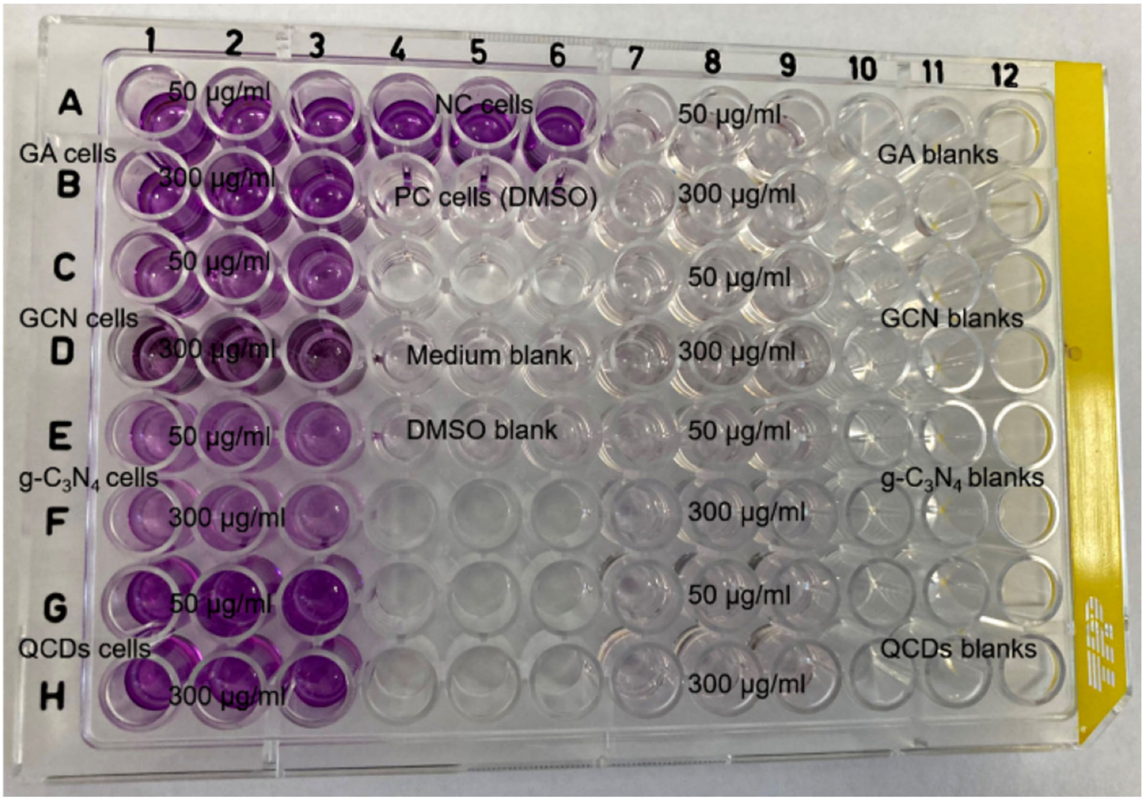

Figure S4. Image of the whole 96-well plate of BJ cells after 24 h incubations with 50 and 300 µg/ml of carbon nanomaterials (g-C_3N_4, QCDs, GA and GCN) immediately before MTT measurement.

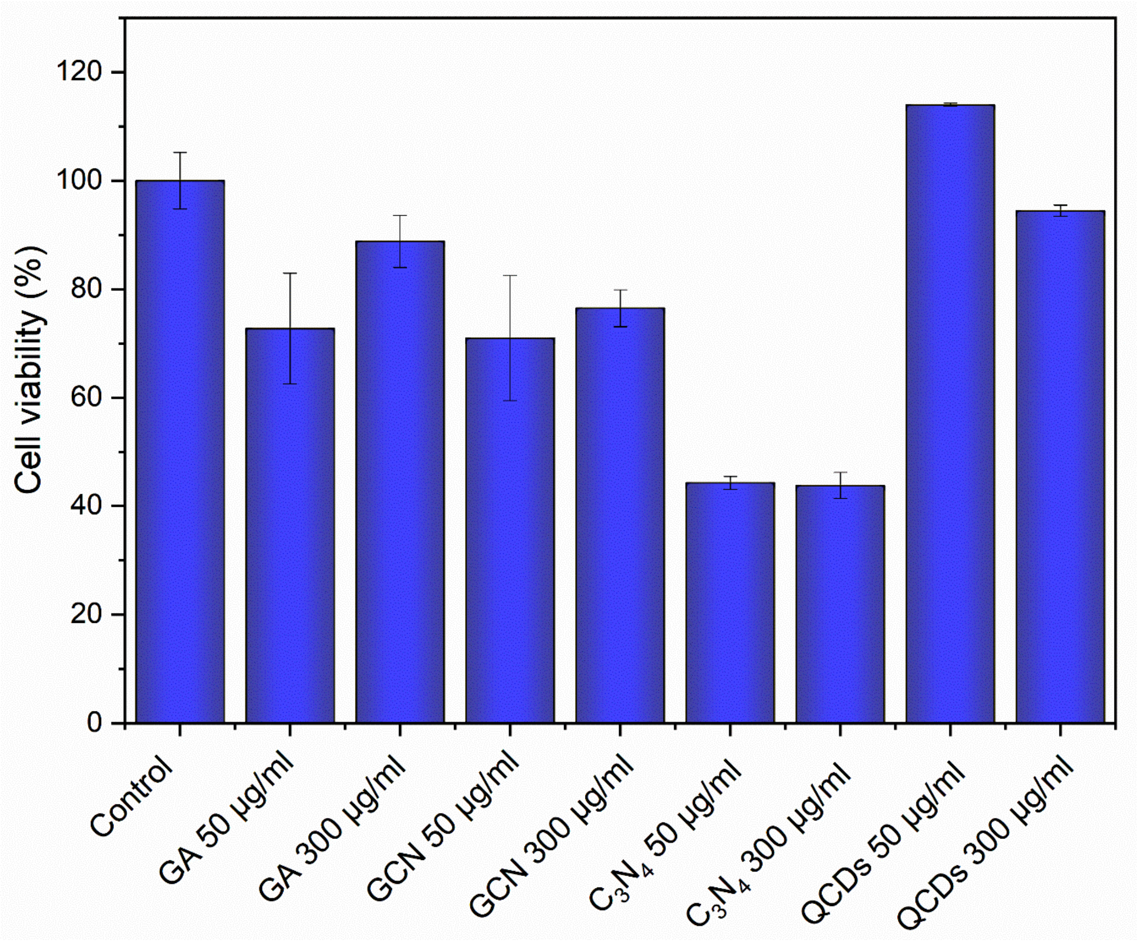

Figure S5. MTT assay results of BJ cells treated with 50 and 300 μg/ml of CNMs for 24 h.

Dot plot of BJ cells treated with g-C_3N_4 sample

As the treatment of the g-C_3N_4 sample caused the BJ cells forward and the side scatter profile to shift out of scale, it was impossible for us to gate the cells for LIVE/DEAD analysis. Therefore, we had to use a dot plot profile of forward scatter values (FSC intensity using linear scale – 0, 50, 100, 150, 200, 250 RFU) against values of detector (blue FL 1: ex.405/em. 528 nm intensity using logarithmic scale – 0, 10^2, 10^3, 10^4, 10^5 RFU) where no increase in the fluorescence intensity was expected (Figure S4). This way, we were able to gate only the population of cells for the analysis.

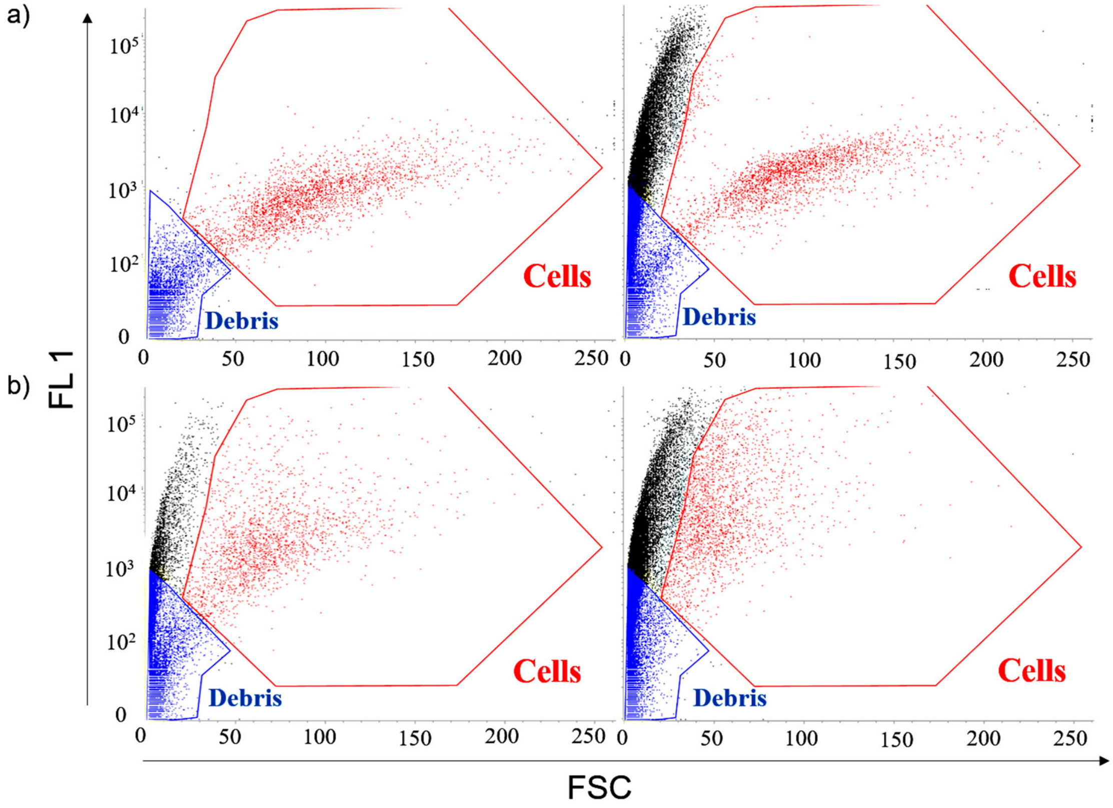

Figure S6. Forward scatter (FSC – A) against fluorescence detector (FL 1) dot plot profiles for samples: a) negative control (left) and g-C_3N_4 spike-in control (right); b) cells treated with 50 µg/ml (left) and 300 µg/ml (right) of g-C_3N_4. The population of cells is highlighted in red, the population of debris is marked blue and the remaining black population in samples g-C_3N_4 and QCDs is considered as NMs agglomerates.

Spike-in positive controls (PC) samples

To better distinguish between the population of dead cells and population of events representing free agglomerates of NMs, we introduced also spike-in samples of positive controls—Spike-in PC. We added volume of NMs stock solutions corresponding to the concentration of 300 µg/ml right before the measurement of the heat-killed cells. Dot plots showing LIVE/DEAD assays of these samples are displayed in Figure S5.

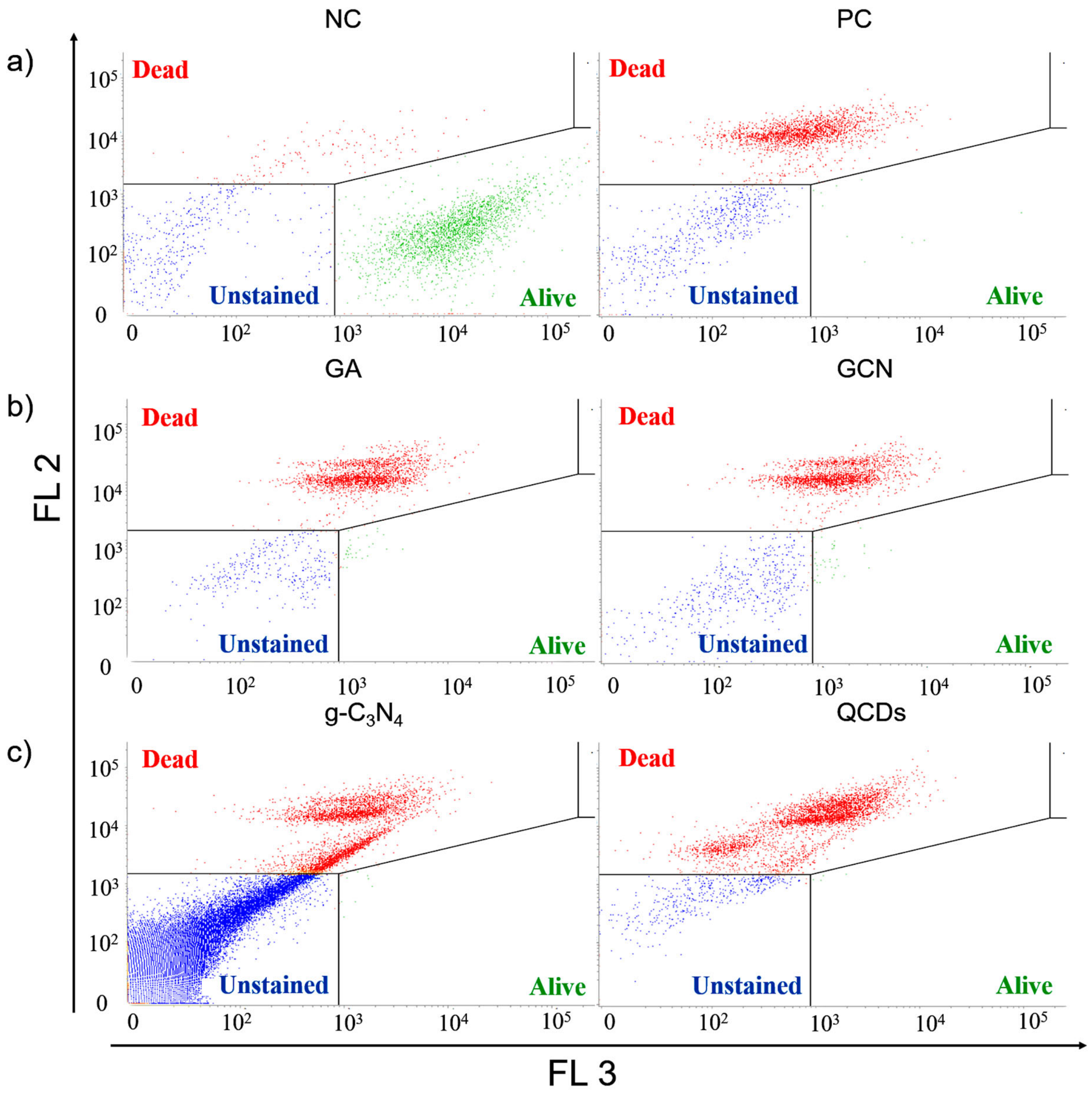

Figure S7. Dot plot showing LIVE/DEAD assay of: a) negative and positive control of BJ cells; b) BJ cells spike-in PC for GA (left) and GCN (right) and c) BJ cells spike-in PC for g-C_3N_4 (left) and QCDs (right). Gates were selected according to the NC and PC samples.

Interference of GCN sample in LIVE/DEAD assay

The GCN sample was in many ways similar to the GA. Treatment with GCN also caused a shift in the scatter profile, but the population of cells was still distinguishable and easy to gate (Figure 2). In addition, microscopy observation was comparable to GA as well, as there were only small

agglomerates, but many NMs were either internalized or attached to the cells’ membranes (Figure S2 and S3).

The only notable difference in the behavior of GCN was that the quenching effect was even stronger than for GA, as interference in the NM PC 300 µg/ml sample led to a higher false increase in viability to 95 %, when standard gating was applied (Figure S8e). Nevertheless, through the new gating protocol according to additional controls, we were still able to overcome this more profound interference (Figure S8f-j) and correct viabilities of BJ cells treated with 50 µg/ml and 300 µg/ml of GCN were 96 and 93 %, respectively (Figure S8j).

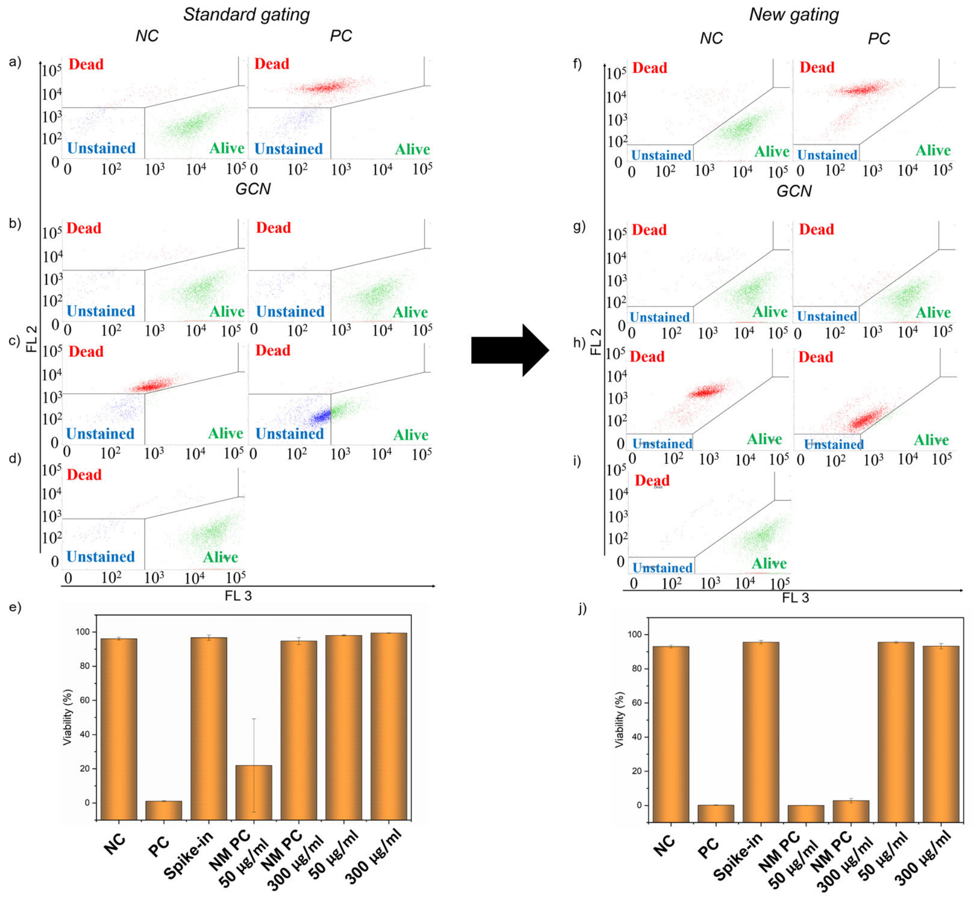

Figure S8. Dot plot showing LIVE/DEAD assay of: (a, f) negative and positive control of BJ cells and (b-d, g-i) BJ cells treated with GCN samples with (a-d) standard gating and (f-i) new gating approach. b, g) Samples treated with 50 µg/ml (left) and 300 µg/ml (right) of GCN for 24 h; c, h) Samples treated with NM PC 50 µg/ml (left) and NM PC 300 µg/ml (right); d, i)

Spike-in control for GCN. e, j). The evaluation of viability of BJ cells treated with GCN and additional control samples (n = 3). The events in *alive* gate are shown in green, events in *dead* gate in red and events in *unstained* gate are highlighted in blue.
